# A Philosopher

**Published:** 1869-05

**Authors:** 


### A PHILOSOPHER.

Mr. J. R. Pierson is a philosopher, because he is a subscriber to Hall's Journal of Health, which is approved and known of all. He had not received his February Journal as soon as he thought he ought to have done, as was the case with some others; but he does not

Rear and tear,

And pull his hair, but draws it very mildly, thus:

"W.W. Hall, M.D. *Sir:*—Allow me to say, in the mildest manner possible, that I have not received the February number. I have two of the bound volumes, and they are very interesting, but I want some new ones, and that is what I sent my dollar and a half for last December."

Also, Mr. Thomas Fool, who writes a splendid hand, says:

"I saw a notice in some paper that Hall's Journal of Health was published for one dollar and a half a year; the money was sent, and I have never received any paper for the same; if the advertisement was not a swindle, I want my paper or my money."

We wish to inform the world at large that publishers of magazines and newspapers are accommodating and liberal always. When their subscribers fail to receive a number, they only require to be notified of the fact promptly, and a duplicate is sent; but our country friends should bear in mind

1st. That there is only one safe way of sending money by mail, and that is by a post office order or other check; and that, if knowing this, they will persist in sending money in letters sometimes unsealed, sometimes with no name, sometimes with no address, it will sometimes happen that the money will spill out by the way, or stick to the fingers of some one who is for the moment oblivious of the law of "meum and tuum."

2d. Sometimes the mails are delayed by snow, or consumed by fire, and now and then a paper goes to a wrong town or State; more than one hundred thousand letters are sent to the dead letter post-office every year, because they are either without a name or an address. It is a very common thing for publishers to receive letters with such omissions; and in such cases can do no less than pocket the money.

3d. Printers cannot be always working; they must have a change; they must be amused; at one time they go on a strike and won't work for love or money, at another they go on a bender.

Our correspondent is a man of the world; he is a business man; he looks on both sides of the question, and brings into view all the possible contingencies of the case.

